# Prognostic Value of Serial Alkaline Phosphatase-to-Albumin Ratio for Mortality After Transjugular Intrahepatic Portosystemic Shunt

**DOI:** 10.3390/medicina62102004

**Published:** 2026-10-16

**Authors:** Sadik Yavuz, Ezgi Comoglu, Derya Ari, Mustafa Ozdemir, Mustafa Comoglu, Meral Akdogan Kayhan

**Affiliations:** 1 Department of Gastroenterology, Ankara Bilkent City Hospital, Ankara 06800, Türkiye; 2 Department of Internal Medicine, Ankara Bilkent City Hospital, Ankara 06800, Türkiye; 3 Department of Internal Medicine, Ankara Etlik City Hospital, Ankara 06170, Türkiye; 4 Department of Radiology, Ankara Bilkent City Hospital, Ankara 06800, Türkiye

**Keywords:** alkaline phosphatase-to-albumin ratio, mortality, portal hypertension, transjugular intrahepatic portosystemic shunt, cirrhosis

## Abstract

***Background:*** Prognostic assessment after transjugular intrahepatic portosystemic shunt (TIPS) remains challenging, and currently available scores are based mainly on pre-procedural variables. The alkaline phosphatase-to-albumin ratio (APAR) is a simple laboratory-derived marker that may provide additional prognostic information during the early post-TIPS period. ***Methods:*** This retrospective, single-center cohort study included 45 adults who underwent TIPS between February 2019 and December 2024. APAR was calculated at baseline, 48 h, and 1 week after the procedure. The primary outcome was one-year all-cause mortality; 90-day mortality was a secondary outcome. Prognostic performance was assessed by receiver operating characteristic (ROC) analysis, and associations with mortality were examined using Cox regression adjusted for age and baseline MELD score. ***Results:*** Within one year, 17 patients (37.8%) died; nine (20.0%) died within 90 days; and 22 (48.9%) died during overall follow-up. Median APAR was higher in patients who died within one year than in those alive at one year at baseline (5.98 vs. 3.89; *p* = 0.006), 48 h (10.79 vs. 3.78; *p* < 0.001), and 1 week (13.93 vs. 4.57; *p* < 0.001). ROC areas under the curve were 0.748 (95% CI, 0.578–0.893), 0.876 (0.748–0.964), and 0.873 (0.726–0.977), respectively. After adjustment for age and MELD, APAR remained associated with one-year mortality at baseline (hazard ratio [HR] 1.140; 95% CI, 1.049–1.238), 48 h (HR 1.225; 1.111–1.350), and 1 week (HR 1.193; 1.092–1.302). ***Conclusions:*** Serial APAR measurements were associated with one-year mortality after TIPS and may support early post-procedural risk assessment. Prospective multicenter validation is needed before clinical implementation.

## 1. Introduction

Portal hypertension is a central feature of decompensated cirrhosis and underlies major complications including variceal bleeding, refractory ascites, and hepatic encephalopathy [1,2,3]. Transjugular intrahepatic portosystemic shunt (TIPS) provides effective portal decompression and is an established treatment for selected patients with recurrent or high-risk variceal bleeding and refractory ascites [4,5]. However, outcomes after TIPS remain heterogeneous. Hepatic encephalopathy, deterioration of hepatic function, and death remain important concerns, making appropriate patient selection and early post-procedural assessment essential [4,6,7].

Several models are used to estimate prognosis in patients undergoing TIPS [8,9]. The Model for End-Stage Liver Disease (MELD) and Child–Pugh scores remain widely used, while the Freiburg Index of Post-TIPS Survival (FIPS), which incorporates age, bilirubin, albumin, and creatinine, was developed specifically for post-TIPS survival prediction [4,8]. Although FIPS improved risk stratification in its derivation cohorts, subsequent validation studies have shown variable discrimination and calibration across different TIPS populations [8,9]. These models are based predominantly on pre-procedural characteristics and may therefore provide limited information on the early physiological response after portal decompression.

TIPS produces abrupt changes in portal and systemic hemodynamics, which may be accompanied by measurable alterations in hepatic and renal biochemical parameters [4,10]. In a study evaluating serial liver tests after TIPS, patients who died within 90 days showed more pronounced and persistent increases in hepatobiliary parameters than survivors, suggesting that early biochemical evolution may carry prognostic information [10]. Serum albumin is also closely related to hepatic functional reserve and nutritional status and may change during the clinical course after TIPS [11]. These observations provide a rationale for evaluating simple laboratory-derived indices during the early post-procedural period.

The alkaline phosphatase-to-albumin ratio (APAR) combines alkaline phosphatase and albumin into a single readily available measure. Higher APAR has recently been associated with increased short- and long-term mortality in critically ill patients with cirrhosis, supporting its potential value as a prognostic marker in advanced liver disease [12]. However, its prognostic role in patients undergoing TIPS has not been established, and the value of serial APAR measurements after the procedure remains unknown. We therefore aimed to evaluate the association of APAR measured before and during the early period after TIPS with mortality and to compare its discriminative performance with established prognostic scores.

## 2. Materials and Methods

### 2.1. Study Design and Population

This retrospective, single-center observational cohort study was conducted in the Department of Gastroenterology at Ankara Bilkent City Hospital. Consecutive adult patients who underwent transjugular intrahepatic portosystemic shunt (TIPS) placement between February 2019 and December 2024 were screened for eligibility. Patients were eligible if they were aged ≥18 years and underwent TIPS for complications of portal hypertension. The indications for TIPS were refractory ascites or variceal bleeding. Patients with malignancy, including hepatocellular carcinoma, acute liver failure, and technically unsuccessful TIPS placement, or insufficient baseline clinical data were excluded. The final study cohort consisted of 45 patients. One-year all-cause mortality following TIPS was assessed in all included patients. The study protocol was approved by the Ankara Bilkent City Hospital Ethics Committee (approval no. TABED 2-24-766) and was conducted in accordance with the principles of the Declaration of Helsinki.

### 2.2. TIPS Procedure and Data Collection

TIPS placement was performed by the interventional radiology team following clinical evaluation and indication by the gastroenterology department. After the procedure, patients were followed according to routine clinical practice, with clinical assessment, laboratory monitoring, and imaging when considered necessary by the treating team. Demographic and clinical data were retrospectively obtained from the hospital electronic medical records and procedural documentation. Recorded baseline variables included age, sex, history of variceal bleeding and hepatic encephalopathy, indication for TIPS, presence of portal vein thrombosis, and spleen size. Additional variables included the underlying etiology of liver disease or portal hypertension, cholestatic and biliary conditions, potential non-hepatic causes of alkaline phosphatase elevation, TIPS procedure type, liver transplantation status, and documented causes of death. Laboratory data were collected at baseline, 48 h, and 1 week after TIPS and included routine hematologic, hepatic, renal, coagulation, and inflammatory parameters. The 48 h measurements were obtained more than 48 h but less than 72 h after TIPS, whereas the 1-week measurements were obtained between days 7 and 10. The Model for End-Stage Liver Disease (MELD), Child–Pugh, Freiburg Index of Post-TIPS Survival (FIPS), albumin–bilirubin (ALBI), and Modified TIPS Score (MOTS) were calculated using baseline data. MOTS was calculated according to the originally described criteria and ranges from 0 to 3 points, with one point assigned for each of the following: INR > 1.6, serum urea > 71 mg/dL, and total bilirubin > 2.2 mg/dL [13].

### 2.3. APAR Calculation and Study Outcomes

The alkaline phosphatase-to-albumin ratio was calculated by dividing serum alkaline phosphatase (U/L) by serum albumin (g/L). APAR was calculated at baseline, 48 h, and 1 week after TIPS using the corresponding laboratory measurements at each time point. The primary outcome was one-year all-cause mortality, defined as death from any cause within 365 days after TIPS. Ninety-day all-cause mortality was evaluated as a secondary outcome. Mortality status was determined from the hospital electronic medical records and the national mortality registration system. Vital status was ascertained through 31 December 2025. Liver transplantation was not considered a censoring event, and follow-up for all-cause mortality continued after transplantation. Follow-up was calculated from the date of TIPS placement to death or the last date of ascertainment. For the primary outcome, patients who survived beyond 365 days were considered alive at one year, regardless of subsequent mortality. Overall mortality during the available follow-up period was also reported descriptively.

### 2.4. Statistical Analysis

Statistical analyses were performed using IBM SPSS Statistics for Windows, version 27.0 (IBM Corp., Armonk, NY, USA), and Python version 3.12 (Python Software Foundation, Beaverton, OR, USA) for survival and prognostic analyses. Continuous variables were summarized as median and interquartile range, while categorical variables were reported as frequency and percentage. Between-group comparisons were performed using the Mann–Whitney U test for continuous variables and Pearson’s chi-square or Fisher’s exact test for categorical variables, as appropriate.

The prognostic performance of APAR for one-year and 90-day all-cause mortality was evaluated using receiver operating characteristic (ROC) analysis at baseline, 48 h, and 1 week. Areas under the curve (AUCs) with 95% confidence intervals were calculated, and optimal cutoff values were determined using the Youden index. Sensitivity and specificity were reported for each threshold. Baseline MELD, Child–Pugh, FIPS, ALBI, and MOTS scores were also assessed by ROC analysis for descriptive comparison. APAR was additionally compared with its individual components, alkaline phosphatase and albumin. Exploratory analyses evaluated the incremental prognostic contribution of APAR to a model including age and baseline MELD score.

Survival was evaluated using the Kaplan–Meier method and Cox proportional hazards regression. Follow-up was restricted to 365 days for the primary outcome and 90 days for the secondary outcome. To minimize guarantee-time bias, landmark analyses were performed at 72 h and day 10 for the 48 h and 1-week APAR measurements, respectively. These analyses included only patients who were alive at the corresponding landmark and had available APAR measurements. Follow-up in each landmark analysis began at the respective landmark and continued until death or the predefined outcome horizon.

Associations between APAR and mortality were examined using Cox proportional hazards regression. Separate analyses were performed for baseline, 48 h, and 1-week APAR. Each time point was first evaluated in an unadjusted model and then in a model adjusted for age and baseline MELD score. Given the cohort size and number of deaths, the adjusted models were deliberately restricted to these covariates to reduce overfitting. Effect estimates are presented as hazard ratios (HRs) with 95% confidence intervals. Cutoff-based survival comparisons and analyses involving limited numbers of events were considered exploratory. All statistical tests were two-sided, and *p* < 0.05 was considered statistically significant.

## 3. Results

### 3.1. Baseline Characteristics

A total of 45 patients who underwent TIPS were included in the analysis, of whom 17 (37.8%) died within one year. The median age of the overall cohort was 54 years (IQR, 41–60), and 30 patients (66.7%) were male. Refractory ascites was the indication for TIPS in 26 patients (57.8%), while 19 patients (42.2%) underwent the procedure for variceal bleeding. Compared with patients alive at one year, those who died within one year were older [58 (50–71) vs. 46.5 (38.75–56.5) years, *p* = 0.023] and had higher baseline APAR values [5.98 (4.42–12.73) vs. 3.89 (2.42–5.01), *p* = 0.006]. Baseline ALP was also higher [229 (109–331) vs. 110.5 (84.5–155.25) U/L, *p* = 0.007]. Child–Pugh scores were higher among patients who died within one year [9 (8–9) vs. 8 (6.75–9), *p* = 0.033]. No significant between-group differences were observed in sex, TIPS indication, portal vein thrombosis, MELD score, Child–Pugh class, FIPS, MOTS, ALBI score, bilirubin, INR, creatinine, sodium, albumin, or CRP. Underlying liver disease etiologies did not differ significantly between groups. Detailed etiologic and hepatobiliary characteristics are provided in Supplementary Table S1. Detailed baseline characteristics are presented in Table 1.

Mortality rates at 30 days, 90 days, and one year after TIPS were 6.7% (n = 3), 20.0% (n = 9), and 37.8% (n = 17), respectively. Overall, 22 patients (48.9%) died during follow-up. Liver failure was the leading cause of death, accounting for 14 of 17 deaths (82.4%) within one year and 18 of 22 deaths (81.8%) during the entire follow-up period. Among patients who underwent TIPS for variceal bleeding, successful hemostasis was achieved in 18 of 19 patients (94.7%). Liver transplantation was documented in 7 of 44 patients (15.5%) with available data. Detailed mortality outcomes and causes of death are presented in Table 2.

### 3.2. Early Post-TIPS Laboratory Changes

APAR remained consistently higher in patients who died within one year during the early post-TIPS period. At 48 h, median APAR was 10.79 (IQR, 5.19–14.64) in patients who died within one year compared with 3.78 (2.64–5.05) in those who survived (*p* < 0.001). At 1 week, the corresponding values were 13.93 (7.07–18.62) and 4.57 (2.78–5.57), respectively (*p* < 0.001). Among the other laboratory parameters, ALP was higher at both post-TIPS time points (both *p* < 0.001). At 48 h, patients who died within one year had higher urea levels (*p* = 0.017) and lower albumin levels (*p* = 0.045). At 1 week, GGT (*p* = 0.008), AST (*p* = 0.003), ALT (*p* = 0.001), and urea (*p* = 0.014) were significantly higher in these patients. No significant differences were observed in creatinine or total bilirubin at either post-TIPS time point. Detailed laboratory findings at each time point are presented in Table 3.

### 3.3. Discriminative Performance of APAR

**Figure 1:**
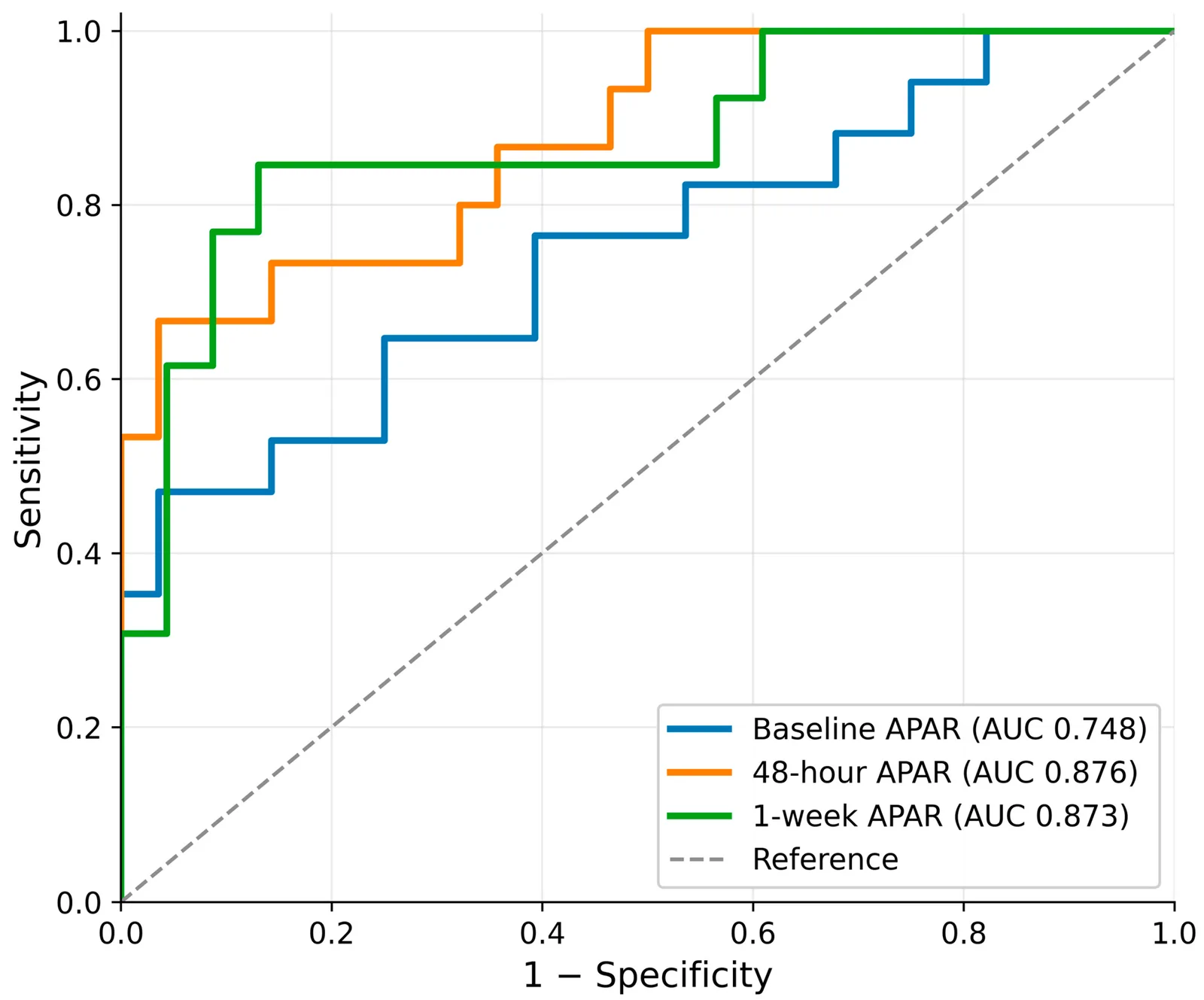
Receiver operating characteristic (ROC) curves of APAR for predicting one-year all-cause mortality at baseline, 48 h, and 1 week after TIPS.

APAR showed good discriminative performance for one-year all-cause mortality at all three assessment time points. Baseline APAR yielded an AUC of 0.748 (95% CI, 0.578–0.893), with an exploratory cutoff of ≥8.00, corresponding to 47.1% sensitivity and 96.4% specificity. At 48 h, the AUC was 0.876 (95% CI, 0.748–0.964); a cutoff of ≥7.34 provided 66.7% sensitivity and 96.4% specificity. At 1 week, the AUC was 0.873 (95% CI, 0.726–0.977), with a cutoff of ≥6.89, 84.6% sensitivity, and 87.0% specificity. For descriptive comparison, the baseline MELD, Child–Pugh, FIPS, ALBI, and MOTS scores showed lower AUCs in this cohort, ranging from 0.520 to 0.685. ROC curves for baseline, 48 h, and 1-week APAR are shown in Figure 1, and detailed diagnostic performance measures are presented in Table 4. APAR showed numerically similar discrimination to ALP at each assessment time, whereas albumin alone had lower AUCs. In an exploratory baseline analysis, adding APAR to a model containing age and MELD increased the apparent AUC from 0.733 to 0.857 (Supplementary Table S2). For 90-day mortality, APAR yielded AUCs of 0.827, 0.875, and 0.856 at baseline, 48 h, and 1 week, respectively (Supplementary Table S3).

### 3.4. Association Between APAR and Mortality

**Figure 2:**
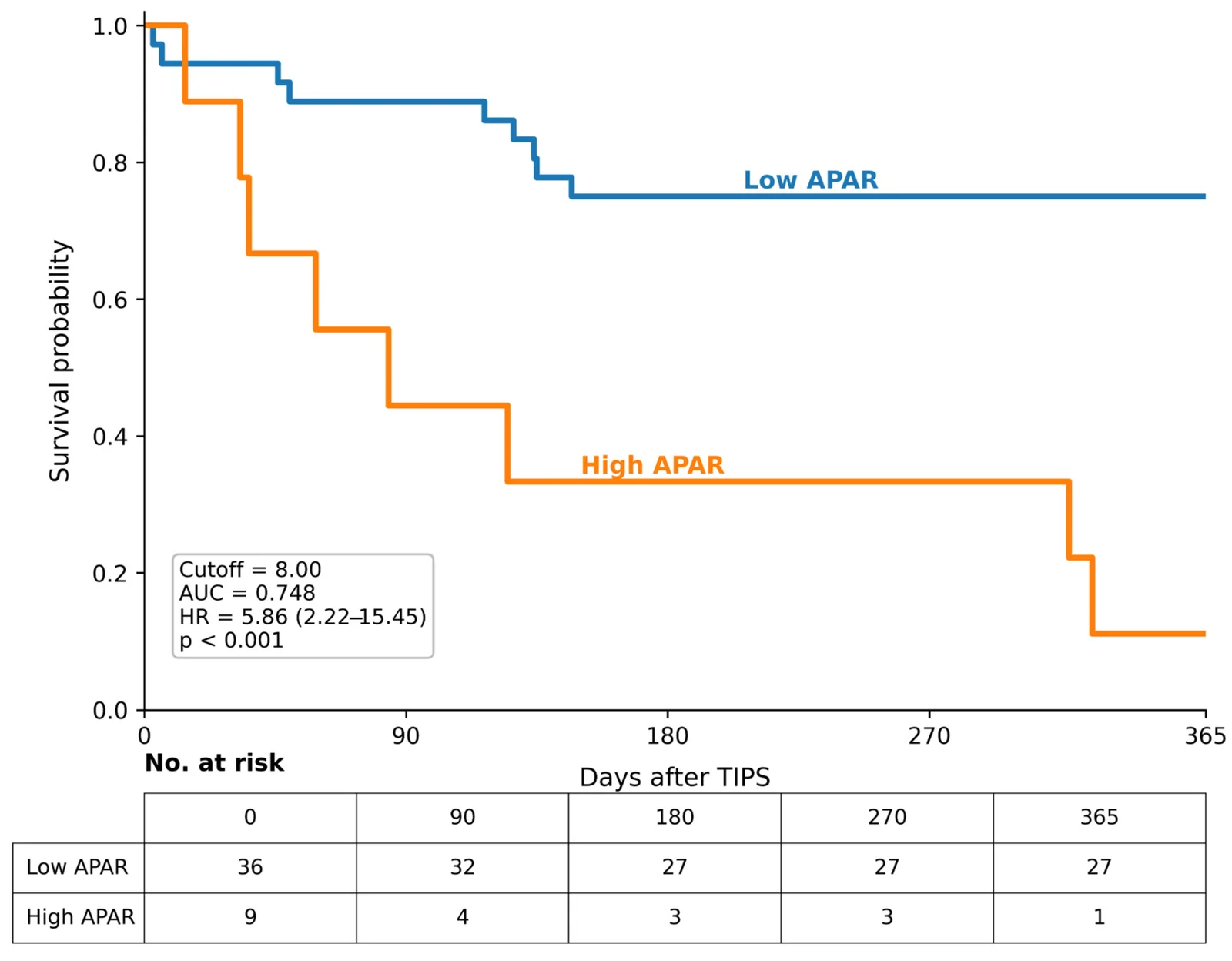
Kaplan–Meier survival curves according to baseline alkaline phosphatase-to-albumin ratio (APAR) in patients undergoing transjugular intrahepatic portosystemic shunt (TIPS).

Higher APAR was associated with an increased hazard of one-year all-cause mortality at all three time points. At baseline, each 1-unit increase in APAR was associated with a higher mortality hazard in the unadjusted Cox model (HR 1.122, 95% CI 1.041–1.208; *p* = 0.002). The association remained significant after adjustment for age and baseline MELD score (adjusted HR 1.140, 95% CI 1.049–1.238; *p* = 0.002). Similarly, 48 h and 1-week APAR remained associated with one-year mortality after adjustment, with HRs of 1.225 (95% CI 1.111–1.350; *p* < 0.001) and 1.193 (95% CI 1.092–1.302; *p* < 0.001), respectively. Significant associations were also observed in the corresponding unadjusted models (Table 5). In the landmark analyses, 43 patients with 15 subsequent one-year deaths were included at 72 h, and 36 patients with 13 deaths were included at day 10 (Supplementary Table S4). For 90-day mortality, the adjusted HRs were 1.140 (95% CI 1.014–1.281; *p* = 0.028), 1.220 (95% CI 1.067–1.395; *p* = 0.004), and 1.230 (95% CI 1.076–1.407; *p* = 0.002) for baseline, 48 h, and 1-week APAR, respectively (Table 5). Kaplan–Meier analysis showed lower one-year survival among patients with baseline APAR ≥ 8.00 compared with those with APAR < 8.00 (unadjusted HR 5.86, 95% CI 2.22–15.45; *p* < 0.001; Figure 2).

In an exploratory sensitivity analysis excluding patients with primary cholestatic disease or biliary obstruction or stricture, baseline APAR remained associated with one-year mortality after adjustment for age and MELD (n = 40; 14 deaths; adjusted HR 1.143, 95% CI 1.037–1.259; *p* = 0.007; Supplementary Table S5).

## 4. Discussion

In this retrospective cohort study of patients undergoing TIPS, APAR was consistently associated with mortality. Non-survivors had higher APAR values before the procedure and during early follow-up, with the strongest discrimination observed at 48 h and 1 week. Importantly, the association between APAR and mortality persisted after adjustment for age and baseline MELD score at each time point. These findings suggest that APAR may provide prognostic information not only before TIPS but also during the early post-procedural period, when changes in clinical and biochemical status may become increasingly relevant.

Risk stratification after TIPS remains challenging, as the performance of available prognostic scores is not uniform across patient populations or follow-up periods [14]. FIPS was developed specifically for patients undergoing TIPS and showed better discrimination than several conventional models in its derivation cohorts, although subsequent external validation demonstrated more modest performance and limitations in calibration [8,9]. Similar variability has been reported in other large cohorts. In 709 patients undergoing TIPS, MELD and MELD-Na performed better than FIPS for short-term mortality, whereas a separate cohort of 855 patients showed improved 3-month prognostic performance with MELD 3.0 [15,16]. A recent meta-analysis likewise found that the relative performance of these models differed according to the time horizon, with MELD performing well for short-term survival and FIPS and MELD-Na showing greater value for longer-term outcomes [14]. In our cohort, the established baseline scores showed limited discrimination, whereas baseline APAR demonstrated a numerically higher AUC. Although this does not establish superiority, it supports further evaluation of complementary markers that may capture prognostic information not fully reflected by conventional baseline scores.

The association between APAR and mortality is biologically plausible. Serum albumin is a well-established marker of hepatic functional reserve and is influenced by nutritional status, systemic inflammation, and circulatory dysfunction in advanced liver disease [17,18]. Its prognostic relevance is reflected in several commonly used scoring systems, including Child–Pugh, ALBI, and FIPS. Alkaline phosphatase is less specific but may reflect hepatobiliary injury and cholestatic burden; higher ALP levels have also been associated with adverse long-term outcomes in cirrhosis [19]. Combining these two routinely available parameters may therefore provide a simple measure that incorporates different aspects of disease severity. Supporting this concept, Peng et al. recently evaluated 2109 critically ill patients with cirrhosis and found a consistent association between higher APAR and increased 28-day, 90-day, and 365-day all-cause mortality after multivariable adjustment [12]. Our findings extend this observation to the TIPS setting, where APAR was associated with mortality before the procedure and remained significant after adjustment for age and baseline MELD score.

Although baseline ALP was significantly higher in patients who died within one year, baseline albumin was numerically lower without reaching statistical significance (28 vs. 30.5 g/L; *p* = 0.108). However, at 48 h, albumin was significantly lower in these patients (25 vs. 28.5 g/L; *p* = 0.045), suggesting that post-procedural albumin levels may also be associated with mortality. Albumin reflects hepatic synthetic reserve, nutritional status, and systemic inflammation, providing a biological rationale for its inclusion in APAR. The limited sample size may have reduced the ability to detect an association with baseline albumin. Nevertheless, APAR and ALP alone showed similar discrimination at baseline (AUC 0.748 vs. 0.742), while a model containing ALP and albumin as separate variables yielded an AUC of 0.739. Interestingly, adding APAR to a clinical model containing age and MELD increased the apparent AUC from 0.733 to 0.857 (Supplementary Table S2). Although this finding suggests potential additional prognostic information beyond these clinical variables, it does not establish superiority over ALP alone and requires validation in larger cohorts.

APAR measured after TIPS also showed good discrimination in the landmark analyses, although direct comparisons of AUCs across time points are limited by differences in the analyzed populations. The association between post-procedural APAR and mortality is biologically plausible because TIPS produces immediate changes in portal and systemic hemodynamics, and the biochemical response during the first days may reflect how well the patient tolerates portal decompression [20]. Earlier work showed that patients who died within 90 days after TIPS had more pronounced and persistent elevations in hepatobiliary laboratory parameters than survivors, with abnormalities lasting beyond the first week in those with poor outcomes [10]. More recent prospective data also support the prognostic value of early post-procedural changes: Reuken et al. found that the evolution of portal pressure gradient within 24 h after TIPS was independently associated with 90-day and 1-year survival [21]. Current EASL guidance likewise recommends early post-TIPS monitoring of transaminases, cholestatic parameters, coagulation indices, and blood counts to detect clinically relevant deterioration [5]. Taken together, these data suggest that post-procedural measurements may complement baseline risk assessment by capturing the early physiological response to TIPS. In this context, the good discrimination observed with 48 h APAR in our cohort is particularly noteworthy because it can be obtained early and from routinely available laboratory tests.

From a clinical perspective, APAR has the advantage of being derived from two inexpensive and routinely available laboratory parameters, without requiring additional testing or complex calculations. In our cohort, 48 h APAR provided good discrimination for one-year mortality, with an AUC of 0.876 and a cutoff of ≥7.34 yielding 66.7% sensitivity and 96.4% specificity. The 1-week value showed similarly good discrimination, although its later availability and the smaller number of patients with measurements limit its immediate clinical applicability. These findings suggest that APAR may be more useful as a complementary dynamic marker during early post-TIPS follow-up than as a replacement for established pre-procedural scores. This approach is consistent with the growing emphasis on reassessing risk after TIPS rather than relying solely on baseline characteristics [10,21]. Nevertheless, the cutoff values identified in our cohort should be regarded as exploratory and require validation in larger, independent populations before they can be incorporated into clinical decision-making.

The present study has several limitations. First, its retrospective design carries the inherent risk of selection bias and unmeasured confounding. Second, the relatively small sample size may have reduced the precision of the estimates and limits the generalizability of the findings. Additionally, information on post-TIPS administration of intravenous albumin, plasma, and blood products was not systematically available. These interventions may have influenced serum albumin concentrations and consequently APAR values. Pre- and post-TIPS portal pressure gradient measurements were also unavailable, preventing assessment of their potential influence on the observed association between APAR and mortality. Third, the APAR cutoff values were derived and evaluated within the same cohort and should therefore be regarded as exploratory rather than definitive clinical thresholds. Finally, although the regression models were adjusted for age and baseline MELD score, the limited number of outcome events, particularly in the 90-day analyses, increased the risk of overfitting and precluded more extensive multivariable adjustment. Despite the sensitivity analysis excluding documented cholestatic or biliary conditions, residual confounding by underlying liver disease etiology or unrecognized cholestatic pathology cannot be excluded. Furthermore, a non-hepatic contribution to ALP elevation cannot be definitively excluded without isoenzyme testing. Landmark analyses also cannot entirely eliminate potential selection bias related to unavailable post-TIPS laboratory measurements.

## 5. Conclusions

In conclusion, APAR may represent a simple and readily available marker for risk assessment in patients undergoing TIPS, with particular potential during early post-procedural follow-up. Its calculation from routinely obtained laboratory parameters may facilitate repeated assessment without additional testing or cost. Future prospective, multicenter studies with larger populations and external validation are needed to confirm its prognostic utility and to clarify whether serial APAR assessment can add clinically meaningful information to established risk stratification strategies.

## Figures and Tables

**Table 1:** Baseline characteristics of the study population according to one-year all-cause mortality.

Variable	Overall (*n* = 45)	Alive at 1 Year (*n* = 28)	Died Within 1 Year (*n* = 17)	*p* Value
Age, years	54 (41–60)	46.5 (38.75–56.5)	58 (50–71)	0.023
Male sex, *n* (%)	30 (66.7)	20 (71.4)	10 (58.8)	0.517
Previous variceal bleeding, *n* (%)	23 (51.1)	16 (57.1)	7 (41.2)	0.365
Previous hepatic encephalopathy, *n* (%)	14 (31.1)	7 (25.0)	7 (41.2)	0.326
TIPS indication, *n* (%)				0.222
Refractory ascites	26 (57.8)	14 (50.0)	12 (70.6)	
Variceal bleeding	19 (42.2)	14 (50.0)	5 (29.4)	
Emergency/rescue TIPS, *n* (%)	4 (8.9)	2 (7.1)	2 (11.8)	0.626
Listed for liver transplantation, *n* (%)	27 (60.0)	18 (64.3)	9 (52.9)	0.537
Portal vein thrombosis, *n* (%)	10 (22.2)	7 (25.0)	3 (17.6)	0.719
Spleen size, mm	164 (135–190)	176 (129.5–196.25)	145 (135–174)	0.347
Underlying etiology, *n* (%)				0.669
MASLD/MASH	13 (28.9)	7 (25.0)	6 (35.3)	
Alcohol-related	6 (13.3)	3 (10.7)	3 (17.6)	
Viral hepatitis (HBV/HCV)	8 (17.8)	5 (17.9)	3 (17.6)	
Other etiologies	18 (40.0)	13 (46.4)	5 (29.4)	
MELD score	16 (11–20)	15 (11–17.25)	18 (10–22)	0.511
Child–Pugh score	8 (8–9)	8 (6.75–9)	9 (8–9)	0.033
Child–Pugh class, *n* (%)				0.058
A	7 (15.6)	7 (25.0)	0 (0.0)	
B	30 (66.7)	16 (57.1)	14 (82.4)	
C	8 (17.8)	5 (17.9)	3 (17.6)	
FIPS	0.02 (−0.46–0.27)	0.07 (−0.47–0.24)	−0.08 (−0.35–0.27)	0.833
MOTS	3 (2–3)	3 (2–3)	3 (2–3)	0.451
ALBI score	−1.54 (−1.91–−1.31)	−1.65 (−2.07–−1.34)	−1.46 (−1.80–−1.20)	0.125
Hemoglobin, g/dL	9.7 (8.5–11.2)	9.7 (8.73–11.25)	9.9 (8.4–11)	0.981
WBC, ×10^9^/L	4.74 (2.57–6.89)	3.83 (2.08–6.46)	4.92 (3.63–7.5)	0.287
Platelets, ×10^9^/L	99 (61–164)	89 (44.25–148)	129 (74–164)	0.287
NLR	4.25 (2.86–6.7)	3.7 (2.71–5.42)	4.7 (3.39–10.22)	0.087
AST, U/L	46 (29–80)	45 (30.5–81)	59 (26–80)	0.806
ALT, U/L	29 (19–46)	29 (22.75–43)	31 (16–48)	0.888
ALP, U/L	124 (99–194)	110.5 (84.5–155.25)	229 (109–331)	0.007
GGT, U/L	67 (31–110)	65 (31–89.5)	71 (37–148)	0.331
Total bilirubin, mg/dL	1.5 (1.13–3.04)	1.59 (1.25–3.07)	1.4 (0.8–2.2)	0.393
Direct bilirubin, mg/dL	0.7 (0.4–1.3)	0.75 (0.47–1.07)	0.7 (0.4–1.3)	0.851
INR	1.3 (1.3–1.5)	1.35 (1.27–1.5)	1.3 (1.3–1.5)	0.924
Urea, mg/dL	50 (30–74)	47 (27.25–57.75)	51 (36–122)	0.092
Creatinine, mg/dL	0.84 (0.69–1)	0.84 (0.69–1)	0.89 (0.75–1.52)	0.393
Sodium, mmol/L	134 (132–139)	136.5 (133–140)	133 (130–135)	0.062
Albumin, g/L	29 (26–33)	30.5 (28–33.25)	28 (25–30)	0.108
CRP, mg/L	9.4 (0.5–26.92)	3.7 (0.95–22.55)	14.8 (0.05–31.5)	0.406
APAR (ALP/albumin ratio)	4.46 (2.95–6.52)	3.89 (2.42–5.01)	5.98 (4.42–12.73)	0.006

Data are presented as median (interquartile range) or n (%). The “Other etiologies” category includes cryptogenic, cholestatic, autoimmune, vascular and miscellaneous causes; detailed etiologies and hepatobiliary characteristics are presented in Supplementary Table S1. Abbreviations: ALBI, albumin–bilirubin; ALP, alkaline phosphatase; ALT, alanine aminotransferase; APAR, alkaline phosphatase-to-albumin ratio; AST, aspartate aminotransferase; CRP, C-reactive protein; FIPS, Freiburg Index of Post-TIPS Survival; GGT, gamma-glutamyl transferase; INR, international normalized ratio; MASLD, metabolic dysfunction-associated steatotic liver disease; MASH, metabolic dysfunction-associated steatohepatitis; MELD, Model for End-Stage Liver Disease; MOTS, Modified TIPS Score; NLR, neutrophil-to-lymphocyte ratio; TIPS, transjugular intrahepatic portosystemic shunt; WBC, white blood cell count.

**Table 2:** Mortality outcomes and causes of death following TIPS.

Variable	Overall Cohort (*n* = 45)	Died Within 1 Year (*n* = 17)	All Deaths (*n* = 22)
Mortality outcomes			
30-day mortality, *n* (%)	3 (6.7)		
90-day mortality, *n* (%)	9 (20.0)		
1-year mortality, *n* (%)	17 (37.8)		
Overall mortality, *n* (%)	22 (48.9)		
Time to death, days, median (IQR)		84 (36–134)	126 (47–324)
Causes of death, *n* (% of deaths)			
Liver failure		14 (82.4)	18 (81.8)
Variceal bleeding		1 (5.9)	1 (4.5)
Sepsis/infection		1 (5.9)	2 (9.1)
Cardiovascular cause		1 (5.9)	1 (4.5)
Variceal bleeding control (*n* = 19)			
Successful hemostasis, *n* (%)	18 (94.7)		
Hemostasis failure, *n* (%)	1 (5.3)		
Documented liver transplantation, *n* (%)	7 (15.5)		

Data are presented as *n* (%) or median (interquartile range). Mortality percentages refer to all 45 patients; cause-of-death percentages refer to deaths within 1 year (*n* = 17) or during total follow-up (*n* = 22). Time to death was calculated from TIPS placement. Bleeding-control percentages refer to 19 patients with a variceal bleeding indication. Abbreviations: IQR, interquartile range; TIPS, transjugular intrahepatic portosystemic shunt.

**Table 3:** Early post-TIPS laboratory parameters according to one-year mortality.

Variable	Time Point	Alive at 1 Year	Died Within 1 Year	*p*
APAR	Baseline	3.89 (2.42–5.01)	5.98 (4.42–12.73)	0.006
	48 h	3.78 (2.64–5.05)	10.79 (5.19–14.64)	<0.001
	1 week	4.57 (2.78–5.57)	13.93 (7.07–18.62)	<0.001
ALP, U/L	Baseline	110.5 (84.5–155.25)	229 (109–331)	0.007
	48 h	111.5 (89.25–144.25)	275.5 (138–359.75)	<0.001
	1 week	127 (100.5–143.5)	353 (193–425)	<0.001
Albumin, g/L	Baseline	30.5 (28–33.25)	28 (25–30)	0.108
	48 h	28.5 (25–34)	25 (23–30)	0.045
	1 week	28 (26–33)	25.5 (23.25–29)	0.124
GGT, U/L	Baseline	65 (31–89.5)	71 (37–148)	0.331
	48 h	60 (39–90.5)	95.5 (63.25–143.5)	0.081
	1 week	71 (50.5–98)	150 (106–213)	0.008
AST, U/L	Baseline	45 (30.5–81)	59 (26–80)	0.806
	48 h	65 (45.75–76.25)	65 (55–125)	0.251
	1 week	40 (31–58)	108 (77.25–129.5)	0.003
ALT, U/L	Baseline	29 (22.75–43)	31 (16–48)	0.888
	48 h	40 (23–51.5)	34 (27–54)	0.851
	1 week	28 (21–43.5)	63 (42–77.25)	0.001
Total bilirubin, mg/dL	Baseline	1.59 (1.25–3.07)	1.40 (0.80–2.20)	0.393
	48 h	1.98 (1.31–3.41)	1.00 (0.94–2.66)	0.153
	1 week	2.44 (1.55–3.18)	1.92 (1.29–7.15)	0.938
Creatinine, mg/dL	Baseline	0.84 (0.69–1.00)	0.89 (0.75–1.52)	0.393
	48 h	0.80 (0.67–0.94)	0.95 (0.73–1.43)	0.119
	1 week	0.90 (0.75–1.07)	0.85 (0.73–1.48)	0.605
Urea, mg/dL	Baseline	47 (27.25–57.75)	51 (36–122)	0.092
	48 h	36 (24–50)	58 (36–117)	0.017
	1 week	34 (28.5–60.5)	52 (41.25–94)	0.014

Data are presented as median (interquartile range). APAR was calculated as alkaline phosphatase (U/L) divided by albumin (g/L). For APAR, the numbers of patients who died within one year and those alive at one year were 17/28 at baseline, 15/28 at 48 h, and 13/23 at 1 week, respectively. Abbreviations: ALP, alkaline phosphatase; ALT, alanine aminotransferase; APAR, alkaline phosphatase-to-albumin ratio; AST, aspartate aminotransferase; GGT, gamma-glutamyl transferase; TIPS, transjugular intrahepatic portosystemic shunt.

**Table 4:** Discriminative performance of APAR, its individual components, and established prognostic scores for one-year mortality after TIPS.

Predictor	N/Deaths	AUC	95% CI	Optimal Cutoff	Sensitivity, %	Specificity, %
APAR and constituent laboratory markers						
Baseline APAR	45/17	0.748	0.578–0.893	≥8.00	47.1	96.4
48 h APAR	43/15	0.876	0.748–0.964	≥7.34	66.7	96.4
1 week APAR	36/13	0.873	0.726–0.977	≥6.89	84.6	87.0
Baseline ALP	45/17	0.742	0.565–0.887	≥229	52.9	96.4
48 h ALP	43/15	0.860	0.726–0.960	≥235	66.7	96.4
1 week ALP	36/13	0.863	0.702–0.983	≥172	84.6	87.0
Baseline Albumin	45/17	0.645	0.471–0.817	≤26	47.1	82.1
48 h Albumin	43/15	0.686	0.509–0.842	≤32	100.0	32.1
1 week Albumin	36/13	0.676	0.488–0.846	≤24	53.8	82.6
Established baseline prognostic scores						
MELD score	45/17	0.560	0.371–0.756	≥18	52.9	75.0
Child–Pugh score	45/17	0.685	0.539–0.826	≥9	64.7	67.9
FIPS	45/17	0.520	0.340–0.685	≥−0.35	76.5	39.3
ALBI score	45/17	0.639	0.467–0.805	≥−1.91	94.1	35.7
MOTS	45/17	0.559	0.403–0.712	≤1	17.6	100.0

Data are apparent AUCs for death within 365 days after TIPS. AUC 95% CIs were calculated by 2000 stratified bootstrap resamples. N/deaths denotes patients analyzed/deaths by one year. Post-TIPS samples were obtained >48 to <72 h (“48 h”) and on days 7–10 (“1 week”); only patients alive at the day-3 and day-10 landmarks, respectively, with APAR available were evaluated. Thresholds were selected using the Youden index in the same cohort and are exploratory; lower albumin and MOTS were oriented toward higher risk. Abbreviations: ALBI, albumin–bilirubin; ALP, alkaline phosphatase; APAR, alkaline phosphatase-to-albumin ratio; AUC, area under the receiver operating characteristic curve; CI, confidence interval; FIPS, Freiburg Index of Post-TIPS Survival; MELD, Model for End-Stage Liver Disease; MOTS, Modified TIPS Score.

**Table 5:** Cox regression analyses of APAR for one-year and 90-day all-cause mortality after TIPS.

APAR Time Point	N/Deaths	Model	HR	95% CI	*p* Value
One-year all-cause mortality					
Baseline	45/17	Unadjusted	1.122	1.041–1.208	0.002
	45/17	Adjusted for age and MELD	1.140	1.049–1.238	0.002
48 h	43/15	Unadjusted	1.219	1.116–1.331	<0.001
	43/15	Adjusted for age and MELD	1.225	1.111–1.350	<0.001
1 week	36/13	Unadjusted	1.182	1.090–1.282	<0.001
	36/13	Adjusted for age and MELD	1.193	1.092–1.302	<0.001
90-day all-cause mortality					
Baseline	45/9	Unadjusted	1.125	1.013–1.250	0.028
	45/9	Adjusted for age and MELD	1.140	1.014–1.281	0.028
48 h	43/8	Unadjusted	1.227	1.080–1.394	0.002
	43/8	Adjusted for age and MELD	1.220	1.067–1.395	0.004
1 week	36/6	Unadjusted	1.233	1.079–1.409	0.002
	36/6	Adjusted for age and MELD	1.230	1.076–1.407	0.002

Values are hazard ratios (HRs) per 1-unit increase in APAR, with 95% confidence intervals (CIs). N/deaths indicates analyzed patients and subsequent deaths before the specified horizon (90 or 365 days after TIPS). Baseline follow-up begins on the TIPS date; laboratory measurement windows were >48 to <72 h (“48 h”) and days 7–10 (“1 week”), with Cox landmark follow-up beginning at day 3 and day 10, respectively. For all models, follow-up ends by day 90 or day 365 after TIPS. The 90-day adjusted analyses, particularly the one-week model with only 6 events, are exploratory and at high risk of overfitting. Abbreviations: APAR, alkaline phosphatase-to-albumin ratio; MELD, Model for End-Stage Liver Disease; TIPS, transjugular intrahepatic portosystemic shunt.

## Data Availability

The data presented in this study are available from the corresponding author upon reasonable request. The data are not publicly available due to patient privacy and ethical restrictions.

## Supplementary Materials

The following supporting information can be downloaded at: https://www.mdpi.com/article/10.3390/medicina62102004/s1. Table S1: Detailed Etiology and Hepatobiliary Characteristics According to One-Year Mortality; Table S2: Comparative Discrimination of APAR, ALP, and Albumin for One-Year Mortality; Table S3: Discriminative Performance of Laboratory Markers and Prognostic Scores for 90-Day Mortality; Table S4: Landmark Eligibility and Availability of Serial APAR Measurements; Table S5: Sensitivity Analysis Excluding Cholestatic and Biliary Conditions.
