# Supplementary material for: Prognostic Value of Serial Alkaline Phosphatase-to-Albumin Ratio for Mortality After Transjugular Intrahepatic Portosystemic Shunt
Source: Medicina (Kaunas). 2026 Oct 16;62(10):2004. doi: 10.3390/medicina62102004 (PMC13706896; doi:10.3390/medicina62102004)
Supplement: Supplementary file 1 [file medicina-62-02004-s001.zip › medicina-4544221-supplementary.pdf]

Supplementary Table S1. Detailed Etiology and Hepatobiliary Characteristics According to One-Year Mortality

Variable	Overall (n = 45)	No one-year death (n = 28)	One-year death (n = 17)	p
**Liver disease / portal hypertension etiology**				
MASLD/MASH	13 (28.9)	7 (25.0)	6 (35.3)	—
Alcohol-related	6 (13.3)	3 (10.7)	3 (17.6)	—
Hepatitis B	6 (13.3)	5 (17.9)	1 (5.9)	—
Hepatitis C	2 (4.4)	0 (0.0)	2 (11.8)	—
Cryptogenic	7 (15.6)	7 (25.0)	0 (0.0)	—
Budd–Chiari syndrome	3 (6.7)	3 (10.7)	0 (0.0)	—
Primary sclerosing cholangitis	2 (4.4)	0 (0.0)	2 (11.8)	—
AIH–PBC overlap	1 (2.2)	1 (3.6)	0 (0.0)	—
PBC	1 (2.2)	0 (0.0)	1 (5.9)	—
Autoimmune hepatitis	1 (2.2)	0 (0.0)	1 (5.9)	—
Portal vein thrombosis	1 (2.2)	1 (3.6)	0 (0.0)	—
Wilson disease	1 (2.2)	1 (3.6)	0 (0.0)	—
Cardiac-related	1 (2.2)	0 (0.0)	1 (5.9)	—
**Hepatobiliary and cholestatic conditions**				
Documented primary cholestatic etiology (PSC, PBC, or AIH–PBC overlap)	4 (8.9)	1 (3.6)	3 (17.6)	0.144
Primary sclerosing cholangitis	2 (4.4)	0 (0.0)	2 (11.8)	—
AIH–PBC overlap	1 (2.2)	1 (3.6)	0 (0.0)	—
PBC	1 (2.2)	0 (0.0)	1 (5.9)	—
Biliary obstruction or stricture	2 (4.4)	1 (3.6)	1 (5.9)	1.000
Active cholangitis	0 (0.0)	0 (0.0)	0 (0.0)	—
Known non-hepatic source of ALP elevation	0 (0.0)	0 (0.0)	0 (0.0)	—
**Additional clinical context**				
Elective TIPS	41 (91.1)	26 (92.9)	15 (88.2)	0.626
Listed for transplantation	27 (60.0)	18 (64.3)	9 (52.9)	0.537

Values are n (%), with percentages calculated within each column. Abbreviations: AIH, autoimmune hepatitis; ALP, alkaline phosphatase; PBC, primary biliary cholangitis; PSC, primary sclerosing cholangitis; TIPS, transjugular intrahepatic portosystemic shunt.

Supplementary Table S2. Comparative Discrimination of APAR, ALP, and Albumin for One-Year Mortality

**Assessment time**	**Marker**	**Patients / deaths**	**AUC (95% bootstrap CI)**
Baseline	APAR	45/17	0.748 (0.578–0.893)
Baseline	ALP	45/17	0.742 (0.565–0.887)
Baseline	Albumin	45/17	0.645 (0.471–0.817)
48 h (72-h landmark)	APAR	43/15	0.876 (0.748–0.964)
48 h (72-h landmark)	ALP	43/15	0.860 (0.726–0.960)
48 h (72-h landmark)	Albumin	43/15	0.686 (0.509–0.842)
1 week (day-10 landmark)	APAR	36/13	0.873 (0.726–0.977)
1 week (day-10 landmark)	ALP	36/13	0.863 (0.702–0.983)
1 week (day-10 landmark)	Albumin	36/13	0.676 (0.488–0.846)
**Exploratory baseline Cox model comparisons**			
Baseline	APAR alone	45/17	0.748 (apparent)
Baseline	ALP alone	45/17	0.742 (apparent)
Baseline	Albumin alone	45/17	0.645 (apparent)
Baseline	ALP + albumin	45/17	0.739 (apparent)
Baseline	Age + MELD	45/17	0.733 (apparent)
Baseline	Age + MELD + APAR	45/17	0.857 (apparent)

AUCs were calculated in the same eligible patients within each measurement window. Baseline: TIPS date; 48-h samples: >48–<72 h, with survival analysis landmark at 72 h; one-week samples: days 7–10, with landmark at day 10. The outcome was death by 365 days after TIPS. Albumin was evaluated in the inverse direction (lower values indicating greater risk). CIs were generated by stratified bootstrap resampling (2000 iterations). These apparent AUCs should not be interpreted as evidence that APAR is superior to ALP or albumin. The Cox-model AUCs are in-sample (without optimism correction); exploratory nested model comparison of age + MELD versus age + MELD + APAR: likelihood-ratio chi-square = 7.79, p = 0.005.

Supplementary Table S3. Discriminative Performance of Laboratory Markers and Prognostic Scores for 90-Day Mortality

Assessment time	Predictor	Patients / deaths	AUC (95% bootstrap CI)	Exploratory cutoff	Sensitivity (%)	Specificity (%)
Baseline	APAR	45/9	0.827 (0.694–0.938)	≥5.00	88.9	72.2
Baseline	ALP	45/9	0.802 (0.636–0.941)	≥229.00	66.7	88.9
Baseline	Albumin	45/9	0.667 (0.444–0.849)	≤29.00	88.9	55.6
48 h (72-h landmark)	APAR	43/8	0.875 (0.754–0.971)	≥6.55	87.5	77.1
48 h (72-h landmark)	ALP	43/8	0.832 (0.657–0.971)	≥235.00	75.0	85.7
48 h (72-h landmark)	Albumin	43/8	0.704 (0.493–0.893)	≤23.00	50.0	91.4
1 week (day-10 landmark)	APAR	36/6	0.856 (0.605–1.000)	≥14.66	83.3	93.3
1 week (day-10 landmark)	ALP	36/6	0.822 (0.533–1.000)	≥353.00	83.3	90.0
1 week (day-10 landmark)	Albumin	36/6	0.639 (0.403–0.839)	≤24.00	66.7	76.7
Baseline	MELD	45/9	0.534 (0.259–0.789)	≥18.00	55.6	69.4
Baseline	Child–Pugh	45/9	0.616 (0.443–0.781)	≥8.00	100.0	22.2
Baseline	FIPS	45/9	0.449 (0.228–0.696)	≥0.73	22.2	97.2
Baseline	ALBI	45/9	0.647 (0.423–0.860)	≥-1.31	55.6	77.8
Baseline	MOTS	45/9	0.485 (0.313–0.682)	≤1.00	11.1	94.4

AUCs reflect mortality within 90 days of the TIPS procedure. Post-TIPS analyses use the 72-hour and day-10 landmarks, as described in Supplementary Table S4. Albumin and MOTS were oriented so that greater modeled risk corresponds to mortality. Cutoffs were selected in the same cohort using the Youden index and are exploratory. Bootstrap confidence intervals used 2000 stratified replicates. Estimates from different landmarks involve different at-risk populations and should not be directly compared as if based on identical patients.

Supplementary Table S4. Landmark Eligibility and Availability of Serial APAR Measurements

APAR assessment	Landmark (day)	Alive at landmark	APAR available	APAR unavailable	Deaths by landmark	Deaths by day 90*	Deaths by day 365*
Baseline	0	45	45	0	0	9	17
48 h (72-h landmark)	3	44	43	1	1	8	15
1 week (day-10 landmark)	10	43	36	7	2	6	13

*Laboratory samples were obtained before TIPS (baseline), >48 to <72 h after TIPS ("48 h"), or between days 7 and 10 ("1 week"). To avoid guarantee-time bias, follow-up for post-TIPS associations begins at 72 h and day 10, respectively, among patients alive beyond the landmark and with the relevant APAR measurement. *Deaths by day 90 or day 365 among patients eligible and with APAR available; events occur after the corresponding landmark. Absence of a laboratory value does not imply death. Individual reasons for unavailable tests were not coded in the available dataset. APAR, alkaline phosphatase-to-albumin ratio.*

Supplementary Table S5. Sensitivity Analysis Excluding Cholestatic and Biliary Conditions

**Analysis population**	**N / deaths**	**APAR AUC (95% CI)**	**Unadjusted HR (95% CI)**	**p**	**Adjusted HR (95% CI)**	**p**
Full cohort	45 / 17	0.748 (0.578–0.893)	1.122 (1.041–1.208)	0.002	1.140 (1.049–1.238)	0.002
Excluded primary cholestatic etiologies	41 / 14	0.725 (0.545–0.886)	1.119 (1.032–1.213)	0.006	1.144 (1.039–1.260)	0.006
Excluded cholestatic etiologies, biliary obstruction/stricture, or active cholangitis	40 / 14	0.714 (0.533–0.879)	1.116 (1.029–1.210)	0.008	1.143 (1.037–1.259)	0.007

Values refer to one-year all-cause mortality after TIPS. Hazard ratios are per 1-unit increase in baseline APAR. Adjusted Cox models included age and baseline MELD score. Primary cholestatic etiologies comprised PSC (n=2), PBC (n=1), and AIH–PBC overlap (n=1). Biliary obstruction/stricture was present in two patients, including one with PSC; there were no patients with documented active cholangitis. Thus, five unique patients were excluded in the most restrictive analysis. AUC confidence intervals were estimated from 2000 stratified bootstrap resamples; the full-cohort CI is as reported in the main analysis. These small-sample sensitivity analyses are exploratory. Abbreviations: AIH, autoimmune hepatitis; ALP, alkaline phosphatase; APAR, alkaline phosphatase-to-albumin ratio; AUC, area under the ROC curve; CI, confidence interval; HR, hazard ratio; MELD, Model for End-Stage Liver Disease; PBC, primary biliary cholangitis; PSC, primary sclerosing cholangitis; TIPS, transjugular intrahepatic portosystemic shunt.
